# *Trypanosoma brucei* DHFR-TS Revisited: Characterisation of a Bifunctional and Highly Unstable Recombinant Dihydrofolate Reductase-Thymidylate Synthase

**DOI:** 10.1371/journal.pntd.0004714

**Published:** 2016-05-13

**Authors:** Marc W. Gibson, Simon Dewar, Han B. Ong, Natasha Sienkiewicz, Alan H. Fairlamb

**Affiliations:** Division of Biological Chemistry and Drug Discovery, School of Life Sciences, University of Dundee, Dundee, United Kingdom; New York University School of Medicine, UNITED STATES

## Abstract

Bifunctional dihydrofolate reductase–thymidylate synthase (DHFR-TS) is a chemically and genetically validated target in African trypanosomes, causative agents of sleeping sickness in humans and nagana in cattle. Here we report the kinetic properties and sensitivity of recombinant enzyme to a range of lipophilic and classical antifolate drugs. The purified recombinant enzyme, expressed as a fusion protein with elongation factor Ts (Tsf) in ThyA^-^ *Escherichia coli*, retains DHFR activity, but lacks any TS activity. TS activity was found to be extremely unstable (half-life of 28 s) following desalting of clarified bacterial lysates to remove small molecules. Stability could be improved 700-fold by inclusion of dUMP, but not by other pyrimidine or purine (deoxy)-nucleosides or nucleotides. Inclusion of dUMP during purification proved insufficient to prevent inactivation during the purification procedure. Methotrexate and trimetrexate were the most potent inhibitors of DHFR (*K*_i_ 0.1 and 0.6 nM, respectively) and FdUMP and nolatrexed of TS (*K*_i_ 14 and 39 nM, respectively). All inhibitors showed a marked drop-off in potency of 100- to 1,000-fold against trypanosomes grown in low folate medium lacking thymidine. The most potent inhibitors possessed a terminal glutamate moiety suggesting that transport or subsequent retention by polyglutamylation was important for biological activity. Supplementation of culture medium with folate markedly antagonised the potency of these folate-like inhibitors, as did thymidine in the case of the TS inhibitors raltitrexed and pemetrexed.

## Author Summary

There are few validated and fully characterised targets suitable for drug discovery against African trypanosomes, causative agents of sleeping sickness in humans and nagana in cattle. Here we report the biochemical properties of the bifunctional enzyme, dihydrofolate reductase–thymidylate synthase (DHFR-TS), and its susceptibility to a range of classical inhibitors normally used in the treatment of cancer, bacterial or protozoal infections. Some of these drugs are extremely potent against the isolated enzyme, but much less so against the intact trypanosome. We have found that modulating certain medium components can affect drug sensitivity, presumably by either competition for uptake and competition for the active site of DHFR-TS. In the case of one human TS inhibitor (raltitrexed) the inhibitor is more potent against the intact parasite. We propose that addition of extra glutamic acid residues not only improves retention in the cell, but also increases potency against TS, as it does in human cells.

## Introduction

**Fig 1:**
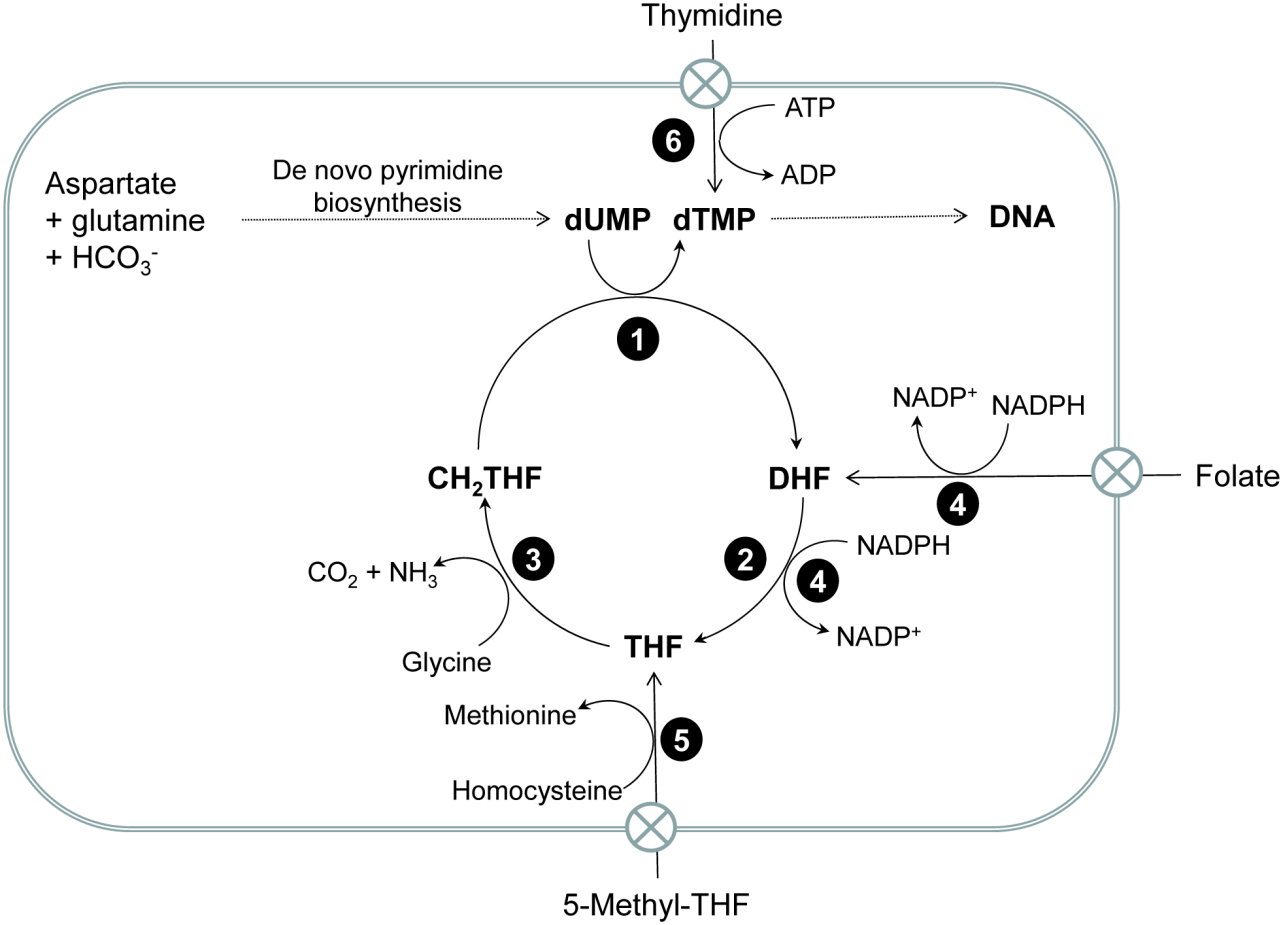
Folate metabolism and the thymidylate cycle in *T*. *brucei*. Enzymes catalysing key metabolic steps are: **1**, thymidylate synthase (EC 2.1.1.45); **2**, dihydrofolate reductase (EC 1.5.1.3); **3**, glycine cleavage system (EC 1.4.4.2, 1.8.1.4 and 2.1.2.10; **4** pteridine reductase (EC 1.5.1.33); **5**, methionine synthase (EC 2.1.1.13); 6, thymidine kinase (EC 2.7.1.21). These parasites are folate and purine auxotrophs and obtain these essential metabolites from the extracellular medium. Although they have retained the capacity for *de novo* pyrimidine biosynthesis, they can also salvage extracellular thymidine, thereby by-passing inhibition of the thymidylate cycle. As reported in this manuscript, folic acid is not a substrate for DHFR, but *Tb*PTR1 is reported to display low activity in reducing folate and dihydrofolate (DHF) [5].

Human African trypanosomiasis (HAT) is an infectious disease caused by two distinct subspecies of the protozoan parasite *Trypanosoma brucei* (*T*. *b*. *gambiense* and *T*. *b*. *rhodesiense*). Existing therapies for this otherwise fatal disease are limited due to toxicity, difficulty in administration, emerging drug resistance and cost. As such, new safe and affordable drugs are required for the continued treatment and control of HAT. Enzymes of essential metabolic pathways in *T*. *brucei*, such as *N*-myristoyltransferase [1] and trypanothione synthetase [2,3], are of continuing interest as novel targets for the development of new treatments, while a number of other putative drug targets remain to be fully exploited. One example is the bifunctional folate and pyrimidine-metabolising enzyme dihydrofolate reductase-thymidylate synthase (DHFR-TS). In *T*. *brucei*, this enzyme is expressed from a single gene as a homodimer comprising of an N-terminal DHFR domain fused via a linker peptide to a TS domain at the C-terminus. In contrast, DHFR and TS are expressed separately from independent genes in many other organisms, including humans. In trypanosomatids, DHFR catalyses reduction of dihydrofolate (DHF) by NADPH to form tetrahydrofolate (THF) which is then converted to *N*^5^, *N*^10^-methylenetetrahydrofolate (CH_2_THF), either via the glycine cleavage system or by serine hydroxymethyltransferase (the latter is absent in *T*. *brucei*). CH_2_THF serves as carbon donor for the reductive methylation of deoxyuridine monophosphate (dUMP) to form thymidylate (dTMP) catalysed by TS [4]. dTMP is ultimately phosphorylated to thymidine triphosphate (dTTP) and used for DNA synthesis and DNA repair (Fig 1). *T*. *brucei* can also salvage extracellular thymidine by-passing *de novo* synthesis. Unlike apicomplexan parasites, trypanosomes lack the ability to synthesise folate and take up serum folate or 5-methyltetrahydrofolate via putative folate transporters. Concentration and retention of folate may involve polyglutamylation as in other organisms, although this has not been established for *T*. *brucei*.

DHFR-TS is essential for cell survival and has been previously validated (both genetically and chemically) as a potential drug target in *T*. *brucei* [6]. Despite significant evolutionary separation between protozoa and mammals, *T*. *brucei* TS is highly similar to its human homologue with 60% identity and an active site that is identical at the amino acid level. *T*. *brucei* DHFR is less well-conserved with only 28% identity with the human enzyme. Indeed, the DHFR domain from several protozoan species has been successfully exploited as a drug target, most notably in the treatment of malaria by the DHF-competitive inhibitors pyrimethamine and cycloguanil [7] which, based on their structural similarity to natural folates, belong to the class of antimetabolites known collectively as the antifolates. These compounds deplete the cellular THF pool, which in turn inhibits dTMP and DNA synthesis resulting in what is known as ‘thymineless-death’ [8,9]. To date, antifolates have not been evaluated as chemotherapeutics in animal models of HAT. Newer antifolates such as nolatrexed [10], pemetrexed [11] and raltitrexed [12] have been designed to directly inhibit TS and have proven useful as cancer chemotherapies; however, these compounds only possess low potency against trypanosomes in thymidine-rich medium [6]. In contrast to *Leishmania* DHFR-TS, the TS domain of *Tb*DHFR-TS has long proven to be an elusive drug target due to an inability to express the enzyme in an active recombinant form [13], which precluded a thorough characterisation of its activity and its sensitivity to inhibitors. Here we describe the first successful recombinant production of bifunctional *T*. *brucei* DHFR-TS (*Tb*DHFR-TS) in the form of a fusion protein incorporating *Escherichia coli* elongation factor Ts (Tsf) [14]. We also biochemically characterise the two activities of *Tb*DHFR-TS and describe their sensitivities to a variety of known inhibitors, along with corresponding *in vivo* potencies in wild type *T*. *brucei*. We show that the challenges faced in recombinant *Tb*DHFR-TS production are the result of instability in the TS domain, rather than proteolysis, as was previously hypothesised [13], and how a combination of TS-stabilising small molecules and macromolecules can overcome this limitation, suggesting that an as-of-yet unidentified TS-stabilising factor could be present in *T*. *brucei* and possibly other species as well. Through comparisons of *in vitro* and *in vivo* potencies of known DHFR and TS inhibitors, we also show that additional targets for these compounds remain to be identified in *T*. *brucei*.

## Methods

### Organisms and reagents

*T*. *brucei* strain 427 was the original source for DNA used in recombinant enzyme production. All reagents were of the highest quality available from Sigma, unless otherwise specified. Recombinant protein expression employed a previously described TS-deficient (*thyA*^-^) *E*. *coli* strain [6], derived from Invitrogen BL21 Star (DE3). Restriction enzymes and *Pfu* DNA polymerase were from Promega. Site-directed mutagenesis was performed using the QuikChange Site-Directed Mutagenesis Kit, Stratagene. DHFR and TS inhibitors were sourced as follows: methotrexate, 5-fluorouracil, 5-fluorodeoxyuridine monophosphate (FdUMP), trimethoprim and pyrimethamine from Sigma Aldrich; nolatrexed, pemetrexed and raltitrexed from Sequoia Research Products; and trimetrexate from Tocris Bioscience.

### Cloning of expression constructs

The solubility enhancing factor Tsf [14] was engineered into a modified pET15b expression vector containing a Tobacco Etch Virus (TEV) protease recognition sequence in place of a thrombin recognition sequence (pET15b*_TEV*) [15]. The *Tsf* open reading frame was amplified by PCR from the genomic DNA (*g*DNA) of *E*. *coli* strain K12 using specific oligonucleotides (*EcTsf*_s and *EcTsf*_as, S1 Table) and *pfu* polymerase. The stop codon in the *Tsf* gene was replaced with a threonine-encoding ACC codon and the PCR product (866 bp) was cloned into the NcoI restriction site on the pET15b*_TEV* vector resulting in an expression cassette containing *Tsf-(His)*_*6*_*-TEV*. The open reading frame of *DHFR-TS* was amplified by PCR from *T*. *brucei g*DNA using specific oligonucleotides (*TbDHFR-TS*_s and *TbDHFR-TS*_as, S1 Table). To express *Tb*DHFR-TS on its own or in frame with *Ec*Tsf, the PCR product (1597 bp) was cloned into the BamHI restriction site in either pET15b*_TEV* or pET15b*_Tsf-TEV* to generate the pET15b*_TEV-DHFR-TS* and pET15b*_Tsf-*TEV-*DHFR-TS* expression constructs, respectively. To create a pET15b_*Tsf*-*TEV*-*TS* fusion construct without the *DHFR* domain, *TS* (884 bp) was PCR-amplified using oligonucleotides *Tb*TS_s and *TbTS*_as (S1 Table) from pET15b_*Tsf-TEV-DHFR-TS* and cloned into the BamHI restriction site on pET15b_*Ts-TEV*. To express DHFR without the TS domain, a stop codon (TAA), immediately after the last amino acid (Arg 239) of DHFR, was introduced into the above DHFR-TS expression constructs (using oligonucleotides *TbDHFR*_mut_s and *TbDHFR*_mut_as, S1 Table) by site-directed mutagenesis (Stratagene), as per manufacturer’s instruction. The accuracy of all constructs was verified by DNA sequencing (http://www.dnaseq.co.uk).

### Recombinant expression and purification of *Tb*DHFR-TS

Expression constructs carrying a TS domain from *T*. *brucei*, *Leishmania major* and human TS (pET15b_*Tsf*-*TEV-TbTS*, pET15b_*Tsf*-*TEV-TbDHFR-TS*, pET15b_*LmDHFR-TS* and pET17b_hTS, respectively) were expressed in a TS-deficient *E*. *coli* strain (*thyA*^-^), while *Tb*DHFR without TS (pET15b_*TEV*-*DHFR*) was expressed in the parental *thyA*^+^ strain. Transformants were selected on LB agar plates containing carbenicillin (50 μg ml^-1^). Plates were initially incubated at 37°C for 18 h and those not displaying colonies were incubated at room temperature for a further 3–6 days until colonies appeared. Single colonies were used to set up starter cultures to inoculate 1 litre of auto-induction media [16] containing 50 μg ml^-1^ carbenicillin. Cultures were incubated at room temperature with shaking at 200 r.p.m for 72 h and aliquots of 50 ml harvested by centrifugation (2,000 g, 10 min, 4°C). Cell pellets were stored at -80°C before use. Pellets were resuspended in lysis buffer (100 mM HEPES, 100 mM NaCl, 1 mM EDTA, 1 mM DTT, pH 7.0), lysed by sonication (3 × 30 s, 10 micron amplitude), clarified by centrifugation (20,000 g, 5 min, 4°C) and supernatants were analysed for DHFR or TS activity.

To purify the recombinant proteins, cultures (1 litre) were harvested by centrifugation (2,800 g, 30 min, 4°C), resuspended in lysis buffer containing cOmplete Protease Inhibitor Cocktail (Roche) and lysed using a cell disruptor (Constant Systems) at 30,000 psi. Lysates were clarified by centrifugation (50,000 g, 30 min, 4°C) and recombinant Tsf-*Tb*DHFR-TS purified using methotrexate affinity chromatography, as previously described [13]. To cleave the Tsf tag, TEV protease was used in a 5:1 (mass to mass) ratio, estimated from DHFR specific activity, at 4°C for up to three days, either in assay buffer following purification or prior to purification in *E*. *coli* lysate treated with up to 40% glycerol. A methotrexate agarose column (5 ml) was loaded by recirculation, monitoring DHFR activity until the column was saturated, and then washed exhaustively with buffers consisting of 50 mM HEPES, 1 M KCl, pH 7, 10% glycerol, followed by 0.5 M KCl, until no further change in absorbance at 280 nM could be detected. Protein was eluted with one column volume of 50 mM HEPES, 0.5 M KCl, pH 8, 10% glycerol with 5 mM DHF. Up to 1 mM dUMP was added to buffers and the column operating temperature reduced to 4°C in an effort to preserve recombinant TS activity. The relative molecular mass of the cleaved recombinant enzyme was determined by size exclusion chromatography on a Superdex 200 column using Bio-Rad gel filtration standards.

### Ethics

All animal experiments were approved by the Ethical Review Committee at the University of Dundee and performed under the Animals (Scientific Procedures) Act 1986 (UK Home Office Project Licence PPL 60/4039) in accordance with the European Communities Council Directive (86/609/EEC).

### Native *T*. *brucei* lysate preparation

*T*. *brucei* trypomastigotes were purified from blood of infected Wistar rats by anion exchange chromatography [17]. Parasites were resuspended (2.5 x 10^9^ cells ml^-1^) in lysis buffer plus cOmplete Protease Inhibitor Cocktail (see above) and biologically inactivated by three rapid freeze-thaw cycles before lysis using a one-shot cell disruptor (Constant Systems) at 30,000 psi. Aliquots (500 μl) were stored at -80°C and clarified by centrifugation (20,000 *g*, 20 min, 4°C) before use.

### Kinetic analysis of DHFR and TS activities

DHFR activity was determined spectrophotometrically at 340 nm [18]. DHFR (5 nM) was pre-incubated with 100 μM NADPH (Medford) in assay buffer (50 mM HEPES, pH 7.4, containing 100 mM KCl) for 1 min at 25°C (1 ml final assay volume), before the addition of 100 μM DHF (Sigma Aldrich). Initial rates were calculated from the combined molar extinction coefficient for NADPH oxidation and DHF reduction (ε = 12,300 M^-1^ cm^-1^). The *K*_m_^app^ values for DHFR substrates were determined by varying the concentration of one substrate in the presence of a fixed saturating concentration of the other. IC_50_ values were determined using 8-point doubling dilutions of inhibitor under the above standard assay conditions.

The initial characterisation of TS was also carried out using a spectrophotometric assay [19,20]. Owing to the pronounced instability of the recombinant protein, clarified *ThyA*^-^ *E*. *coli* lysates were used for characterisation, where the concentration of TS was calculated based on DHFR activity. To determine the *K*_m_^app^ for dUMP, TS (200 nM) was pre-incubated in DHFR-assay buffer containing varying amounts of dUMP (1.56–100 μM) in 1 ml assay volumes. Enzymatic reactions were initiated by the addition of CH_2_THF (200 μM, Shircks Laboratories) and initial rates of CH_2_THF oxidation to DHF monitored by the increase in absorbance at 340 nm (ε = 6,200 M^-1^ cm^-1^). This method is not suitable for determination of the *K*_m_^app^ for CH_2_THF and a radiometric method was used instead [21]. This method measures the release of tritiated water from 5-[^3^H]-dUMP (American Radiochemicals, 14.3 Ci mmol^-1^). Assays (40 μl final volume) contained 200 nM TS and varying amounts of CH_2_THF (37.5 μM– 4.8 mM) in DHFR-assay buffer. Reactions were initiated by adding 200 μM [^3^H]-dUMP (5.55 × 10^5^ dpm nmol^-1^) and stopped after 10 min by the addition of 20 μl trichloroacetic acid. Residual 5-[^3^H]-dUMP was removed by the addition of 200 μl of 10% (w/v) activated charcoal (Sigma). Aliquots (100 μl) of the supernatants were added to 2 ml of scintillation fluid (Pico-Fluor 40^™^, Packard Bioscience) and radioactivity determined using a Beckmann LS 6500 Scintillation Counter. To determine TS activity in clarified *T*. *brucei* lysates the incubation time was increased to 30 min and the assay volume was increased to ~200 μl. To assay overexpressed recombinant activity under comparable linear conditions, working stocks of bacterial lysates were prepared by diluting 20- to 40-fold with 200 μM dUMP, unless otherwise noted. For stability experiments all lysates were desalted using 0.5 ml Zeba Spin Desalting Columns (7K MWCO). Protein concentrations were determined using the BioRad protein assay based on the method of Bradford [22]. Inhibitors (8-point doubling dilutions) were assayed using the radiometric method in the presence of 100 μM CH_2_THF, 200 μM dUMP and 200 nM Tsf-*Tb*DHFR-TS.

Results were analysed by non-linear regression using GraFit v 5.0.13 (Erithacus Software). *K*_m_^app^ values were determined using the Michaelis-Menten equation. IC_50_ values were determined using eq 1 and *K*_i_ values calculated using the Cheng-Prusoff eq 2 [23]. For tight binding inhibitors, where the Hill slope of the IC_50_ equation was > 1, the modified Morrison eq 3 [24,25] was used to calculate *K*_*i*_^app^ to compensate for the effective reduction in total free enzyme concentration.

*Equation 1*. *IC*_*50*_

$$y=\frac{100\%}{1+{\left(\frac{x}{{\text{IC}}_{50}}\right)}^{s}}$$ (1)

(where *y* is the % activity remaining, *x* the inhibitor concentration and *s* the slope factor)

*Equation 2*. *Cheng-Prusoff equation*

$$K_{\text{i}}^{\text{app}}=K_{\text{i}}\left(1+\frac{\left[\text{S}\right]}{K_{\text{m}}}\right)$$ (2)

(where *K*_m_ is the Michaelis-Menten constant, S is the substrate concentration and *K*_i_^app^ is the apparent *K*_i_, expressed here as IC_50_, see eq 1)

*Equation 3*. *Modified Morrison equation for tight-binding inhibition*

$$\frac{v_{\text{i}}}{v_{0}}=1-\frac{\left({\left[\text{E}\right]}_{\text{T}}+{\left[\text{I}\right]}_{\text{T}}+K_{\text{i}}^{\text{app}}\right)-\sqrt{{\left({\left[\text{E}\right]}_{\text{T}}+{\left[\text{I}\right]}_{\text{T}}+K_{\text{i}}^{\text{app}}\right)}^{2}-4{\left[\text{E}\right]}_{\text{T}}{\left[\text{I}\right]}_{\text{T}}}}{2{\left[\text{E}\right]}_{\text{T}}}$$ (3)

(where [E]_T_ is the total enzyme concentration and [I]_T_ the total inhibitor concentration).

### Effects of DHFR and TS inhibitors on growth of wild type *T*. *brucei*

Wild type (WT) *T*. *brucei* bloodstream-form ‘single marker’ S427 were cultured in HMI9T medium [26] supplemented with 2.5 μg ml^-1^ G418 to maintain expression of T7 RNA polymerase and the tetracycline repressor protein [27]. HMI9T medium (standard media for *T*. *brucei* cell culture) contains high concentrations of folate (~9 μM) and thymidine (~160 μM) principally from IMDM and 10% Serum Plus components [6]. A medium based on HMI9T, only lacking Serum Plus, folate and thymidine was prepared in-house, named trypanosome base media (TBM; the residual folate is provided by the serum component). A comparison of these media is described in S2 Table. WT *T*. *brucei* cells grow normally in TBM and the rate of growth is similar to HMI9T in TBM with no supplementation, supplementation with 9 μM folate, 160 μM thymidine or supplementation with both folate and thymidine (7–8 h doubling time).

EC_50_ of antifolates against *T*. *brucei* were determined in 96-well microtitre plates. Serial doubling dilutions of antifolate drugs (10–50 mM stocks prepared in DMSO) were prepared in 100 μl of the appropriate medium and trypanosomes (resuspended in the same medium) added in 100 μl to give a final concentration of 2.5 × 10^3^ cells ml^-1^. All wells, including controls, contained a final volume of 0.5% DMSO. Cultures were incubated for 72 h at 37°C / 5% CO_2_ before cell density was determined using a resazurin-based assay [28]. EC_50_ values were calculated using GraFit v 5.0.13 (Erithacus Software) with a 3-parameter non-linear regression from triplicate readings. Antifolates were tested against parasites cultured in TBM; this allowed for the addition of thymidine (160 μM) and folate (9 μM) respectively.

## Results

### Identification of TS-active recombinant *Tb*DHFR-TS

Initial attempts to express recombinant His_6_-tagged *Tb*DHFR-TS resulted in a low yield of soluble, enzymatically active protein, as previously reported (Table 1) [13]. Although DHFR activity in clarified *E*. *coli* lysates was ~100-fold above background (determined by spectrophotometric assay), the equivalent assay was insufficiently sensitive to detect any TS activity. The functionality of TS could only be confirmed by complementation studies using TS-deficient (*thyA*^-^) *E*. *coli* strain (S1 Fig). ThyA^-^ cells were unable to grow in the absence of thymidine supplementation and growth, albeit very slow, was restored in cells transformed with the pET15b_*Tb*DHFR-TS plasmid with bacterial colonies appearing after 3–6 days incubation. In stark contrast, numerous colonies were formed within 18 h for the positive controls of thyA^+^ *E*. *coli* and thyA^-^ cells complemented with *L*. *major* DHFR-TS (S1 Fig). Re-plating of *Tb*DHFR-TS-complemented cultures resulted in comparable numbers of colonies as seen with positive controls; however, slow growth persisted. These results, together with the undetectable activity of TS in lysates, suggest that the TS domain of *Tb*DHFR-TS could be highly unstable. SDS-PAGE of *E*. *coli* lysates also revealed that the majority of *Tb*DHFR-TS was present in the insoluble pellet of clarified lysates, indicating formation of inclusion bodies.

**Table 1:** Screening for recombinant *T*. *brucei* DHFR and TS activities as expressed in *E*. *coli*.

Construct	N-terminal tag	*E*. *coli* strain	Specific activity, mU mg^-1^		Ratio DHFR:TS
			DHFR	TS	
Tsf blank vector	N/A	*thyA*^-^ ^a^	2.1 ± 0.2	Not detected	—
Tsf blank vector	N/A	*thyA*^+^	3.6 ± 0.2	Not detected ^b^	—
*Tb*DHFR	His_6_	*thyA*^+^	2000 ± 76	Not detected ^b^	—
*Tb*DHFR-TS	His_6_	*thyA*^-^	210 ± 14	Not detected	—
Tsf-*Tb*DHFR-TS	Tsf	*thyA*^-^	1200 ± 7	8.8 ± 0.1	137:1

Enzyme activity was determined in clarified lysates using the spectrophotometric assays described in the methods. Values are the means and standard deviations of triplicate assays from a single experiment.

^a)^ Grown in presence of 50 μg ml^-1^ thymine to compensate for lack of endogenous TS

^b)^ The spectrophotometric assay is insufficiently sensitive to detect endogenous TS activity in the *thyA*^*+*^ *E*. *coli* host

To determine whether the DHFR or TS domain was responsible for the poor solubility, constructs separating the two domains were generated, based upon previously reported boundaries [13]. Expression of the domain-specific constructs resulted in a ~10-fold increased soluble DHFR expression (Table 1), whereas the independent TS domain failed to complement *thyA*^-^ *E*. *coli* (S1 Fig).

Attempts to improve the solubility of TS-active *Tb*DHFR-TS using common fusion partners, such as NusA and thioredoxin, were unsuccessful. As an alternative approach, *E*. *coli* elongation factor Ts (Tsf) was examined as a solubility-enhancer [14]. Tsf was engineered upstream in frame with the His_6_-TEV site within the pET15b expression vector, to generate a *Tsf* expression construct (pET15b_*Tsf*-*His-TEV*). This was used as an expression cassette for the bifunctional DHFR-TS and the individual domains (Tsf-*Tb*DHFR-TS, Tsf-*Tb*TS, Tsf-*Tb*DHFR).

Functional TS activity of Tsf-*Tb*DHFR-TS was confirmed by its ability to complement *thyA*^-^ *E*. *coli* cells, whereas Tsf-*Tb*TS did not (S1 Fig). These results indicate that the DHFR domain provides a structural contribution for TS to be active, which is consistent with previous findings for *Trypanosoma cruzi* DHFR-TS [29] and *Plasmodium falciparum* DHFR-TS [30], suggesting TS is only functional when in complex with DHFR.

### Stabilisation of recombinant *T*. *brucei* TS

DHFR activity in lysates of *E*. *coli* expressing Tsf-*Tb*DHFR-TS was ~6-fold more active than those expressing His_6_-*Tb*DHFR-TS (Table 1). In addition, TS activity which had proved elusive in the His_6_-protein could now be detected. Crucially, the addition of dUMP to assay buffer prior to addition of the enzyme appeared to enhance TS activity. The possible role of dUMP in the stabilisation of TS was therefore further investigated. TS activity in clarified bacterial lysates was stable for up to 72 h at 4°C, whereas less than 5% TS activity was retained if small molecules and metabolites (<1,000 Da) were removed using a desalting spin column. The addition of 200 μM dUMP immediately following desalting preserved the activity of TS, consistent with several other studies reporting substrate-mediated TS stabilisation by dUMP in other organisms [31–34]. In some cases dUMP is reported to have a synergistic action with CH_2_THF; however, in the case of *Tb*DHFR-TS, there was no stabilisation observed with CH_2_THF, possibly due to the low affinity for this substrate (see below). Other pyrimidine nucleotides, including the uracil-containing ribonucleotides and deoxyribonucleotides, and the thymidine-containing deoxyribonucleotides, were also unable to stabilise TS. Furthermore, common stabilisers such as 10% glycerol, 1% BSA and 1 mM EDTA were ineffective in preserving TS activity. To determine if the oxidation of the TS catalytic cysteine could be a reason for its inactivation, 2-mercaptoethanol (10 mM) was tested. However, not only was the reducing agent ineffective, it was found to inhibit TS activity at higher concentrations (EC_50_ ~100 mM), suggesting cysteine oxidation is probably not the cause of TS-inactivation. Thus, preservation of TS activity seemed to be specific for dUMP.

**Fig 2:**
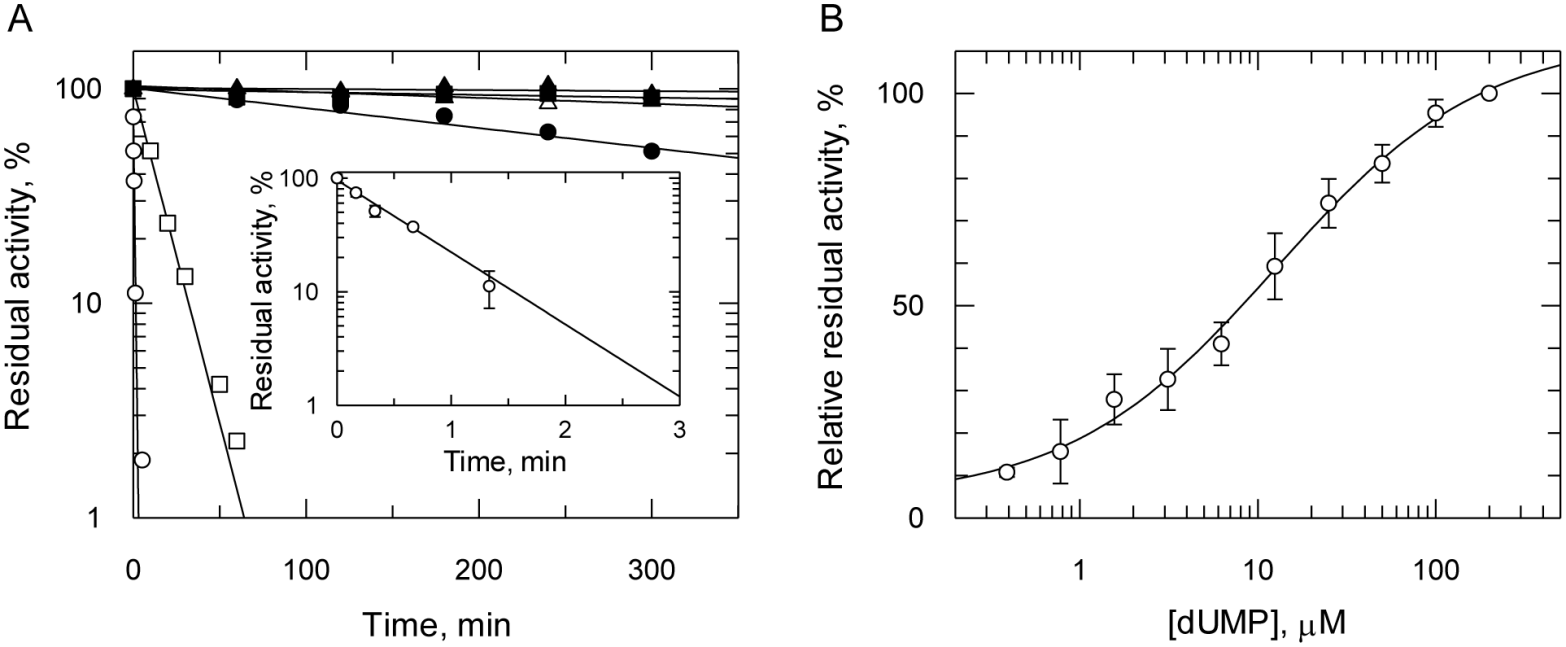
Stabilisation of recombinant *T*. *brucei* TS activity by substrate dUMP. A. Determination of TS stability relative to dUMP concentration using Tsf-*Tb*DHFR-TS from *thyA*^-^ lysate. Tsf-*Tb*DHFR-TS (circles), *Lm*DHFR-TS (squares), human TS (triangles). Incubation mixtures omitted either 200 μM dUMP (open symbols) or 100 μM CH_2_THF (closed symbols); post-incubation, addition of the missing reagent was used to initiate reactions. The *inset* shows the loss of TS activity Tsf-*Tb*DHFR-TS in absence of dUMP over a shorter time scale. All activities were determined in triplicate spectrophotometrically using 100 μM CH_2_THF, as described in the methods. B. Optimum concentration of dUMP required to stabilise the TS activity. Activity was determined by incubating the diluted lysate for 10 min in assay buffer with varying concentrations of dUMP prior to initiation of the reaction with CH_2_THF and excess (200 μM) dUMP.

To characterise the stabilisation of recombinant *T*. *brucei* TS by dUMP, clarified *thyA*^-^ *E*. *coli* lysate containing Tsf-*Tb*DHFR-TS was diluted 100-fold into assay buffer containing 100 μM CH_2_THF and pre-incubated for different times before initiation of the reaction by the addition of dUMP (Fig 2A). In the absence of dUMP following desalting, TS activity decayed rapidly with only 1% residual activity remaining after 3 min. The data was fitted to a single exponential decay yielding a rate constant of 1.46 ± 0.11 min^-1^, from which a half-life of inactivation (t_½_) of 28 ± 2 s can be derived (Fig 2A, inset). The half maximal concentration of dUMP required to stabilise TS activity in the absence of CH_2_THF was 12.6 ± 2.5 μM (Fig 2B). Over longer incubation times the addition of 200 μM dUMP markedly increased the stability of the diluted enzyme by 700-fold (t_½_ = 330 min), but failed to completely stabilise TS activity (Fig 2A).

The stabilising effect of dUMP on Tsf-*Tb*DHFR-TS was compared with *Lm*DHFR-TS and human TS expressed in *thyA*^-^ *E*. *coli* as controls (Fig 2A). Both trypanosomatid enzymes were found to be relatively unstable compared to the mono-functional human TS. Human TS showed little or no activity loss over a five hour period in either the presence or absence of dUMP. In contrast, *Lm*DHFR-TS behaved more like *Tb*DHFR-TS, but was ~20-fold more stable than *Tb*DHFR-TS (t_½_ = 9.7 min). *Tb*DHFR-TS was ultimately only partially stabilised by dUMP, as evidenced by the loss of 50% total activity over a five hour period, whereas *Lm*DHFR-TS was effectively stable in presence of dUMP.

To establish if instability of *Tb*DHFR-TS was also the case with the native enzyme, DHFR and TS activity in lysates of *T*. *brucei* and *thyA*^-^ *E*. *coli* expressing Tsf-*Tb*DHFR-TS were compared (Table 2). Tsf-*Tb*DHFR-TS in desalted lysates is considerably less stable at higher temperature, hence, lysates were incubated at 37°C for 1 h prior to activity determination. Before incubation, the ratio of DHFR- to TS-activity for Tsf-*Tb*DHFR-TS was calculated to be 53:1. Both DHFR and TS activities in the recombinant enzyme decreased drastically following incubation. Once again, the addition of dUMP improved the stability of TS while DHFR activity was unaffected. The ratio of DHFR:TS activity (5:1) of the native enzyme before incubation was in good agreement with values previously reported for *T*. *brucei gambiense* and *T*. *lewisi* lysates [35]. DHFR activity of the native DHFR-TS remained unchanged after incubation, while the decrease in TS activity was comparably less drastic compared to the recombinant enzyme. The addition of dUMP to *T*. *brucei* lysates, however, did not protect against loss of TS activity. These results suggest that the *T*. *brucei* endogenous *Tb*DHFR-TS does not suffer the same inactivation as the recombinant enzyme. In the event that a hitherto unknown TS activating factor might be present in *T*. *brucei* lysate, native and recombinant clarified lysates were combined in a 1:1 ratio; this however resulted in no appreciable improvement in stability.

**Table 2:** Stability of recombinant and native *Tb*DHFR-TS in presence or absence of dUMP in bacterial or trypanosomal lysates.

Sample	DHFR, mU mg^-1^	Residual activity, %	TS mU mg^-1^	Residual activity, %	Ratio DHFR:TS
Tsf-*Tb*DHFR-TS control	1960 ± 168	100	37.3 ± 4.2	100	53
Tsf-*Tb*DHFR-TS, 0.2 mM dUMP	487 ± 65	25	6.30 ± 0.67	17	77
Tsf-*Tb*DHFR-TS, without dUMP	490 ± 30	25	1.66 ± 0.21	4	294
Native enzyme control	4.6 ± 0.8	100	0.95 ± 0.05	100	5
Native enzyme, 0.2 mM dUMP	4.2 ± 0.3	91	0.47 ± 0.05	49	9
Native enzyme, without dUMP	3.4 ± 0.3	74	0.47 ± 0.08	49	7

All samples were desalted and either supplemented to 0.2 mM dUMP or corrected for dilution effect with an equivalent volume of assay buffer prior to incubation at 37°C for 1 h. DHFR activity was determined spectrophotometrically under saturating substrate concentrations and TS by radiometric assay using 2.5 mM CH_2_THF and (saturating) 0.2 mM [3H]-dUMP, as described in the methods section. Control values are activities before incubation. Values are the means and standard deviations of triplicate assays from a single experiment.

### Purification of recombinant *Tb*DHFR-TS

**Fig 3:**
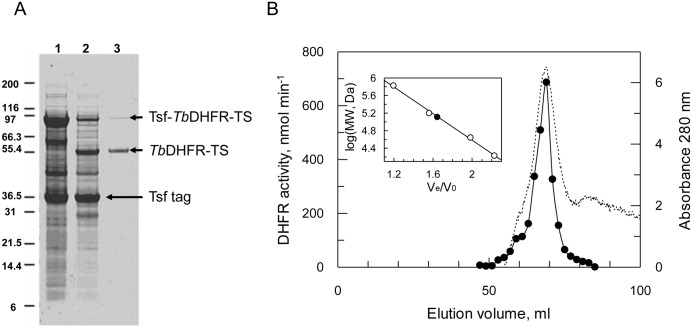
Purification of recombinant Tsf-*Tb*DHFR-TS from *E*. *coli*. A. SDS-PAGE gel stained with Coomassie blue. Lane 1. Clarified *thyA*^-^ *E*. *coli* lysate expressing Tsf-*Tb*DHFR-TS. Lane 2. Glycerol-diluted lysate treated with TEV protease. Lane 3. Methotrexate agarose elution. Proteins indicated by arrows identified by mass spectrometry to be Tsf-*Tb*DHFR-TS, cleaved *Tb*DHFR-TS, and the Tsf tag. B. Size exclusion chromatography of purified product. Closed circles: DHFR activity. Dashed line: absorbance at 280 nm.

Having established the importance of dUMP in stabilising recombinant *Tb*DHFR-TS, it was subsequently included in all buffers used for the purification of Tsf-*Tb*DHFR-TS and its TEV-cleaved counterpart. Despite the presence of dUMP, TS activity was still completely lost during purification regardless of whether TEV cleavage occurred at the start or end of the procedure. Cleavage of Tsf-*Tb*DHFR-TS prior to purification was only possible when glycerol was added as a stabiliser to prevent rapid protein precipitation. Cleaved *Tb*DHFR-TS was then purified by methotrexate agarose affinity chromatography to near homogeneity in ~10% yield, along with some residual un-cleaved Tsf-*Tb*DHFR-TS (Fig 3A). The specific activity for the purified enzyme was 24.3 U mg^-1^ for DHFR, with no detectable TS activity. The cleaved protein behaved as a homodimer on size exclusion chromatography (Fig 3B) and sequence identity confirmed by mass spectrometry fingerprinting with >70% sequence coverage, including the C-terminal amino acid shown to be crucial for TS activity [36]. MALDI-TOF determination of the exact total mass was not possible due to difficulties associated with desorption, although low-resolution data also confirmed the presence of dimeric *Tb*DHFR-TS.

In comparison, affinity chromatography of the un-cleaved Tsf-tagged *Tb*DHFR-TS resulted in a ~7-fold greater yield, suggesting Tsf significantly improved the stability of this enzyme. Following elution from the methotrexate column, some minor contaminating proteins were visible by SDS-PAGE; these were identified by mass spectrometry fingerprinting as Ef-Tu, the binding partner of the Tsf tag, and *E*. *coli* hsp90. The latter could be removed by washing the column with ATP prior to elution with methotrexate. In contrast, the control protein (*Lm*DHFR-TS) could be purified to homogeneity with retention of TS activity (DHFR 21.2 U mg^-1^; TS 0.89 U mg^-1^).

### Kinetic characterisation of DHFR and TS activities

Purified Tsf-*Tb*DHFR-TS was subsequently used for the kinetic characterisation of DHFR domain and clarified crude lysates for the TS domain (S2 Fig). Using the spectrophotometric assay, DHFR displayed a classical bell-shaped pH-optimum profile, with an optimal pH of ~5.5 (S2A Fig). In contrast, TS had a pH optimum of 7.0. The optimal ionic strength for both enzymes required 100 mM KCl, with DHFR displaying 2.5-fold activation and TS 4.5-fold activation (S2B Fig). This is consistent with previously reported KCl-dependent activation of this enzyme, although our pH optimum profiles disagree [13]. TS could also be activated 4.5-fold with 10 mM MgCl_2_ (30 mM ionic strength) and this effect was not additive with activation by KCl. Higher concentrations of MgCl_2_ were inhibitory to TS, consistent with a previous report [37].

**Fig 4:**
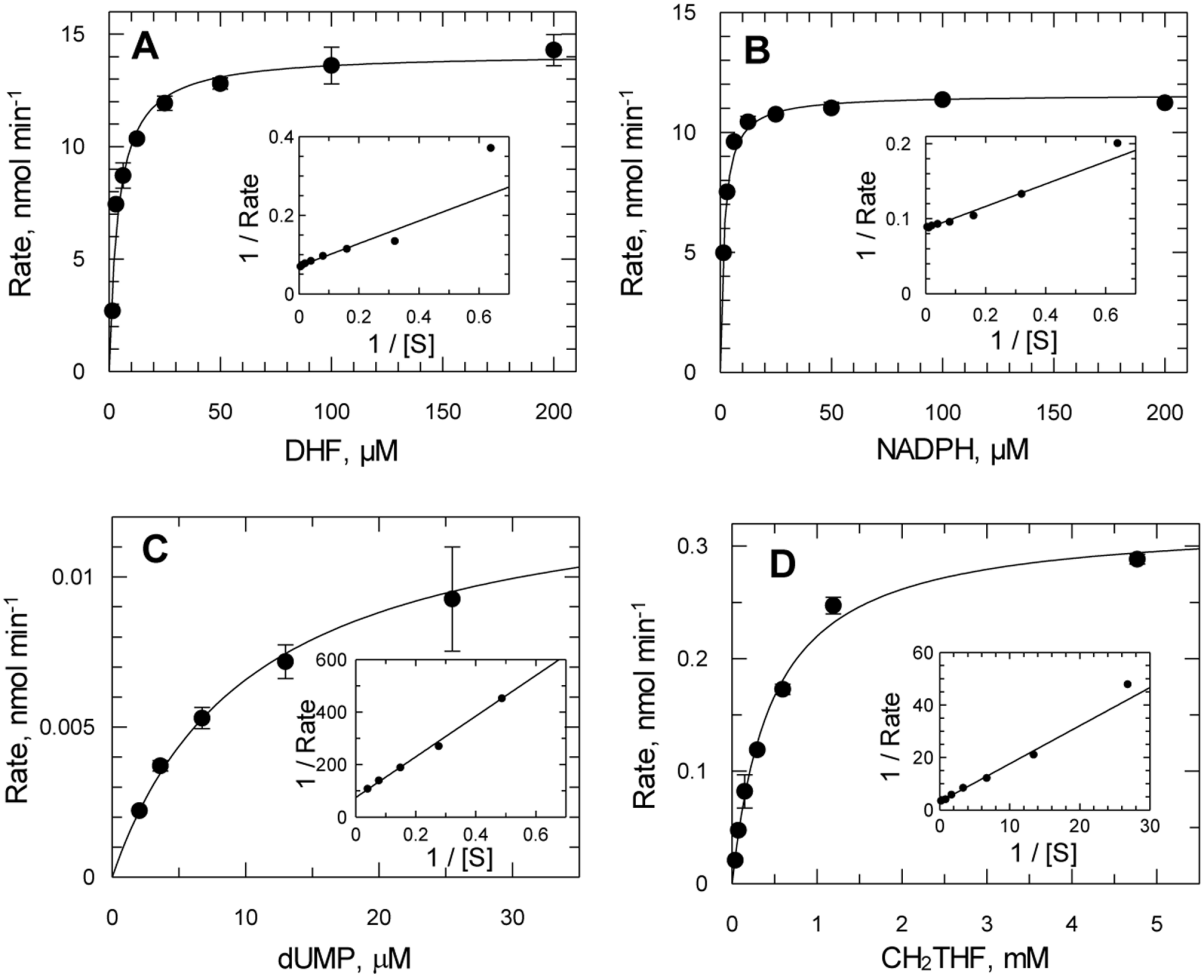
Kinetic constants for recombinant Tsf-*T*bDHFR-TS. (**A**) and (**B**) Determination of DHFR *K*_m_^app^ values for DHF (4.1 ± 0.6 mM) and NADPH (1.7 ± 0.2 mM), respectively, using purified enzyme. (**C**) and (**D**) Determination of TS *K*_m_^app^ values for dUMP (8.2 ± 0.6 mM) and CH_2_THF (470 ± 50 mM), respectively, using clarified *E*. *coli* lysates. Assays were performed as described in methods. Values represent weighted means of three independent determinations. One representative determination is shown for each *K*_m_^app^.

Since the intracellular pH of *T*. *brucei* has been reported to be 7.4 [38], a standardised assay buffer consisting of 50 mM HEPES, pH 7.4 and 100 mM KCl was used for all subsequent studies. Under these conditions, Tsf-*Tb*DHFR-TS obeys simple Michaelis-Menten kinetics with all four substrates (Fig 4). The *K*_m_^app^ of Tsf-*Tb*DHFR-TS and Tsf-cleaved *Tb*DHFR-TS for DHF were identical (4.1 ± 0.6 μM for Tsf-*Tb*DHFR-TS and 4.2 ± 0.5 μM for the cleaved enzyme) demonstrating that the tag did not interfere with the enzyme activity. The catalytic efficiency for reduction of DHF was 6.8 x 10^6^ M^-1^ s^-1^ consistent with DHFR from other organisms (Table 3). Active site titration with methotrexate confirmed DHFR activity corresponding to one site per monomer. Folic acid and the structurally related pterins (biopterin, dihydrobiopterin, sepiapterin and neopterin) were inactive as substrates for *T*. *brucei* DHFR (<17,000-fold and <2,300-fold compared to DHF as substrate for folate and pterins, respectively). The inability of *Tb*DHFR-TS to reduce folate is in agreement with the *L*. *major* enzyme [39]; folate is presumed to be reduced to DHF by PTR1 (Fig 1).

**Table 3:** Kinetic properties of recombinant Tsf-*Tb*DHFR-TS compared with those of related species and the human homologues.

	DHFR					TS				
Species	*k*_cat_, s^-1^	DHF *K*_m_^app^, μM	NADPH *K*_m_^app^, μM	DHF *k*_cat_/*K*_m,_ M^-1^ s^-1^	NADPH *k*_cat_/*K*_m,_ M^-1^ s^-1^	*k*_cat_, s^-1^	CH_2_THF *K*_m_^app^, μM	dUMP *K*_m_^app^, μM	CH_2_THF *k*_cat_/*K*_m,_ M^-1^ s^-1^	dUMP *k*_cat_/*K*_m,_ M^-1^ s^-1^
*T*. *brucei*	26 ± 1^a^	4.1 ± 0.6	1.7 ± 0.2	6.8 × 10^6^	16 × 10^6^	0.7± 0.1^a^	470± 50	8.2± 0.6	1.5 × 10^3^	85 × 10^3^
*T*. *cruzi* ^b^	72	17	1.2	4.2 × 10^6^	60 × 10^6^	3.4	58	1.0	59 × 10^3^	3,400 × 10^3^
*L*. *major* ^c^	27	1.3	0.7	21 × 10^6^	38 × 10^6^	0.44	51	6.0	8.6 × 10^3^	73 × 10^3^
*Crithidia fasciculata* ^d^	12	<2	6.7	6.0 × 10^6^	1.8 × 10^6^	1.4	400	3.3	3.5 × 10^3^	420 × 10^3^
*Plasmodium falciparum* ^e^	33	2.4	5.5	14 × 10^6^	2.5 × 10^6^	3.0	75	7.9	40 × 10^3^	380 × 10^3^
hDHFR ^f^, hTS ^g^,^h^	7.5	2.8	9.1	2.7 × 10^6^	0.82 × 10^6^	0.6	30	1.8	20 × 10^3^	330 × 10^3^

Spectrophotometric assays were used, apart from the determination of *K*_m_^app^ for CH_2_THF which required radiometric assay.

^a)^ Value reported for *Tb*DHFR-TS with Tsf tag removed. Tagged Tsf-*Tb*DHFR-TS *k*_cat_ 17 ± 1 s^-1^. The *k*_cat_ for TS is estimated based on DHFR concentration in parasite lysate, assuming TS full site reactivity.

^b)^ Reche *et al*. [29];

^c)^ Meek *et al*. [40];

^d)^ Ferone and Roland [41];

^e)^ Prapunwattana *et al*. [42];

^f)^ White *et al*. [43];

^g)^. Dolnick and Cheng [32]

^h)^ Reported value is *K*_m_

The *K*_m_^app^ for dUMP (8.2 ± 0.6 μM) is in good agreement with TS from other organisms and is consistent with the half maximal concentration of dUMP (12.6 ± 2.5 μM) required for stability of TS. In contrast, the *K*_m_^app^ values for CH_2_THF were considerably more variable between TS from various organisms. The affinity of the *T*. *brucei* enzyme was more similar to that from the more distantly related *C*. *fasciculata* [37,41] (Table 3). The catalytic efficiency (1.5 x 10^3^ M^-1^ s^-1^) was considerably lower compared to those reported for recombinant *T*. *cruzi* [29] and *L*. *major* [44] enzymes. These discrepancies could be due to the inherent instability of the *T*. *brucei* enzyme or due to competing metabolism of CH_2_THF by other enzymes in the crude *thyA*^-^ *E*. *coli* lysate

### *In vitro* effects of known DHFR and TS inhibitors on *Tb*DHFR-TS

**Fig 5:**
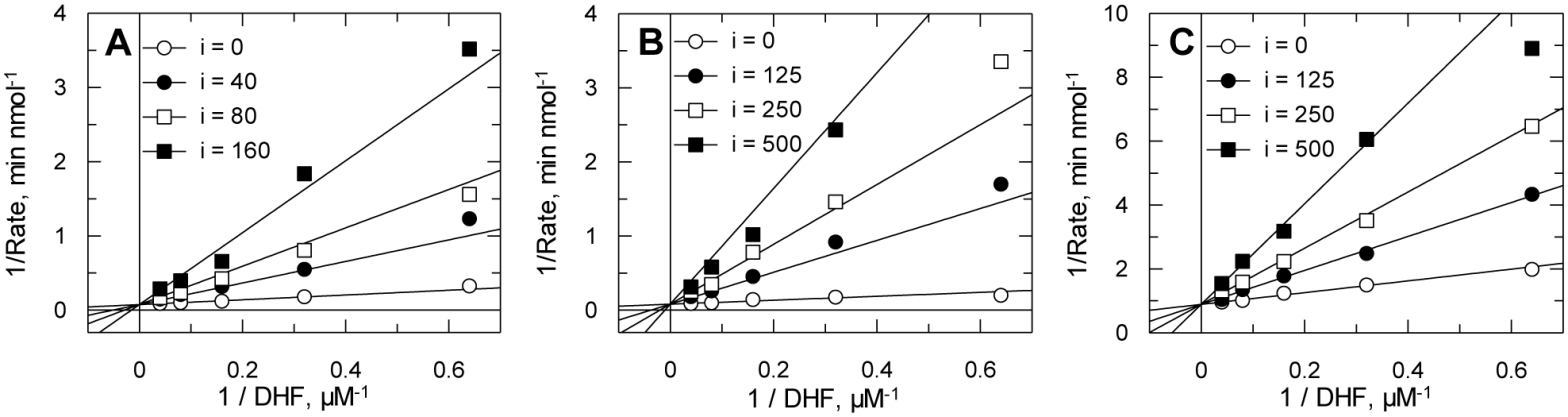
Modality of inhibition of antifolate drugs against DHFR activity. (A) pyrimethamine, (B) trimethoprim and (C) raltitrexed. Recombinant *T*bDHFR-TS was assayed in the presence of varying concentrations of substrate (DHF) and fixed amounts of inhibitor (see legends for concentrations in nM). Each double reciprocal plot was inspected for inhibition pattern and then globally fitted by non-linear regression to equations describing either competitive or mixed inhibition. Statistical F-tests using GraFit revealed that competitive inhibition gave the best fit in all cases.

**Fig 6:**
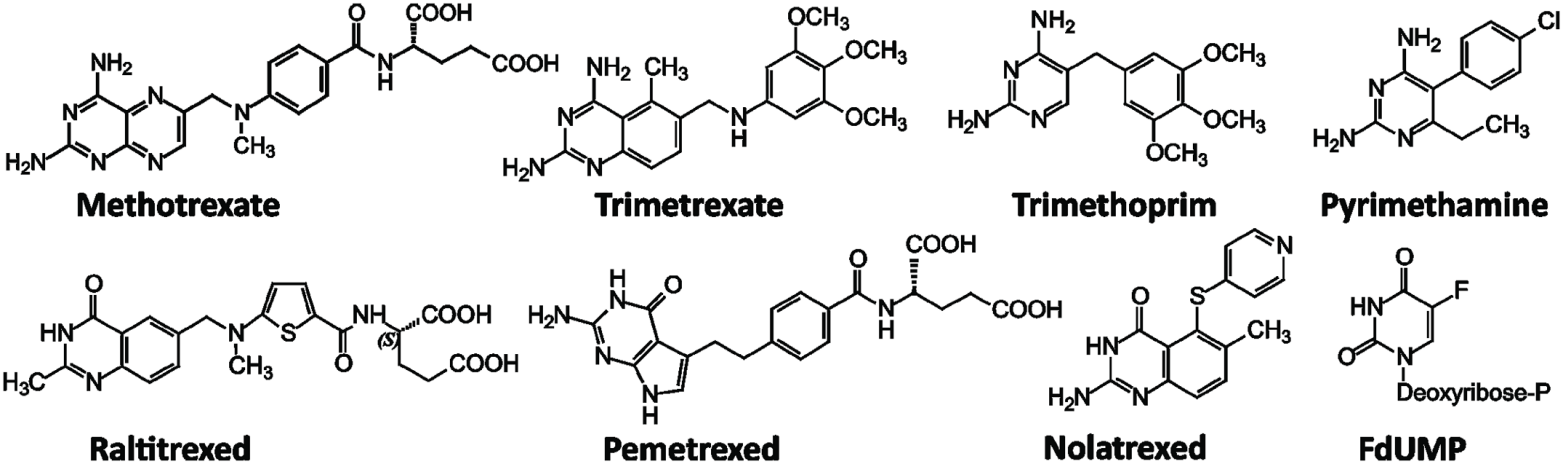
Chemical structures of DHFR and TS inhibitors.

Known inhibitors of DHFR and TS from other organisms were tested for their potencies against recombinant Tsf-*Tb*DHFR-TS (Table 4). The greatest degree of TS inhibition was seen with 5-fluorodeoxyuridine monophosphate (FdUMP), a dUMP-competitive TS-specific inhibitor which displayed tight-binding inhibition. The most potent DHFR inhibitors were the classic antifolate methotrexate and the trimethoxy-substituted trimetrexate. These compounds were found to be tight-binding inhibitors with picomolar *K*_*i*_ values, while other antifolates exhibited linear competitive inhibition with respect to the substrate, DHF. The diaminopyrimidine antifolates trimethoprim and pyrimethamine were found to specifically inhibit DHFR, as did the diaminoquinazoline trimetrexate. Trimethoprim, pyrimethamine and raltitrexed were found to behave as potent competitive inhibitors of DHFR with *K*_*i*_ values of 11.4 ± 1.2, 17.6 ± 2.3 and 70.4 ± 7.2 nM, respectively, (Fig 5). These values are in good agreement with those determined using the IC_50_ method in Table 4. Other antifolates possessed varying degrees of both DHFR and TS inhibition. Apart from the TS substrate analogue FdUMP, the only inhibitor that possessed greater inhibition of TS than DHFR as tested was nolatrexed, showing 10-fold TS selectivity. Chemical structures of the inhibitors are shown in Fig 6.

**Table 4:** Sensitivity of recombinant *Tb*DHFR-TS to DHFR and TS inhibitors compared with their human counterparts.

Inhibitor	DHFR *K*_i_, nM		TS *K*_i_, nM	
	*T*. *brucei*	*Human*	*T*. *brucei*	*Human*
Methotrexate	0.095 ± 0.006	0.0034^a^	46,300 ± 4,800	13,000^g^
Trimetrexate	0.597 ± 0.029	0.040^b^	Inactive *	—
Pyrimethamine	6.35 ± 0.49	1.2^c^ 120^d^	Inactive**	—
Trimethoprim	17.6 ± 1.5	200^c^ 1400^d^	Inactive**	—
Raltitrexed	93.1 ± 7.9	93^e^	215 ± 18	60^e^
Pemetrexed	290 ± 20	7.0^f^	20,500 ± 200	110^f^
Nolatrexed	348 ± 30	Inactive^h^	39.4 ± 4.9	15^h^
FdUMP	Inactive**	Not reported	13.8 ± 1.3	1.7^i^

*K*_*i*_ values were determined using spectrophotometric methods. Values represent weighted mean and standard error of three independent determinations.

**Human data from:**

^a^ Appleman *et al*. [45];

^b^ Jackson *et al*. [46];

^c^ Margosiak *et al*. [47];

^d^ Chowdhury *et al*. [48];

^e^ Jackman *et al*.[12];

^f^ Shih *et al*. [11];

^g^ Allegra *et al*. [49];

^h^ Webber *et al*. [10];

^i^ Dolnick and Cheng [32]

**Inactive:**

* No inhibition at 25 μM;

** No inhibition at 100 μM

### *In vivo* effects of known DHFR and TS inhibitors on *T*. *brucei*

Antifolate drugs had highest potency against bloodstream forms of *T*. *brucei* when tested in a medium deficient in folate and thymidine, with the exception of the lipophilic drug nolatrexed where potency did not change between media types (Table 5). Indeed methotrexate, pemetrexed and raltitrexed possess nanomolar potency in a thymidine and folate deficient media. The addition of folate and thymidine reduced the potencies of the antifolates, except nolatrexed. For methotrexate, pyrimethamine and trimethoprim the addition of folate had a greater effect in reducing potency than the addition of thymidine. For raltitrexed and pemetrexed the addition of thymidine had a greater effect in reducing drug potency than the addition of folate. For the lipophilic inhibitor trimetrexate the addition of folate or the addition of thymidine had a comparable effect on reducing drug potency.

**Table 5:** Effect of folate and thymidine on sensitivity of *T*. *brucei* to DHFR-TS inhibitors *in vitro*.

Inhibitor	No addition	+ Thymidine	+ Folate	+ Thymidine + Folate
Methotrexate	0.012 ± 0.001	0.12 ± 0.03	1.31 ± 0.13	8.16 ± 2.78
Trimetrexate	0.32 ± 0.02	2.82 ± 0.36	1.23 ± 0.06	4.09 ± 0.35
Pyrimethamine	2.26 ± 0.32	2.01 ± 0.34	6.32 ± 0.71	6.87 ± 0.33
Trimethoprim	48.8 ± 3.7	59.8 ± 8.6	170 ± 28	145 ± 22
Raltitrexed	0.038 ± 0.001	> 250	64.8 ± 4.3	> 250
Pemetrexed	0.020 ± 0.001	> 250	12.9 ± 1.1	>250
Nolatrexed	33.8 ± 3.7	32.7 ± 4.7	21.8 ± 2.0	39.6 ± 7.2

Cells were cultured in TBM with the addition of thymidine (160 μM) and/or folate (9 μM) as described in Experimental section. Units for EC_50_ values are μM. Results are the weighted means ± standard error of 3 independent experiments

## Discussion

The TS activity of recombinant *Tb*DHFR-TS is highly unstable (t_½_ 28 s) compared to other organisms, with the *T*. *brucei* enzyme proving to be the least stable TS yet reported. Addition of dUMP increases enzyme stability, as in other organisms, but proved insufficient to achieve purification of active enzyme. Other stabilising agents, including mercaptoethanol, did not prevent inactivation, unlike human TS that can be stored at 4°C for 3 months without loss of activity [50]. This remarkable instability could account for the inefficient complementation and slow growth of TS-deficient *E*. *coli* expressing *Tb*DHFR-TS. However, the basis for instability is not known. A previous report suggested that sequential degradations at the C-terminus together with internal cleavage in the TS domain may be responsible [13]. Our purified recombinant protein showed no evidence of proteolytic cleavage by either SDS-PAGE or MALDI-TOF MS and the C-terminus of TS was identified by MS fingerprinting, including the final residue required for catalysis. Thus, proteolysis can be discounted, as can oxidation of the catalytic cysteine since thiols did not stabilise the protein. Other possible stabilising agents include parasite-specific interacting macromolecules (e.g. mRNA or protein chaperones), or parasite-specific post-translational modifications. Further research is required to test these possibilities. A variety of DHFR and TS inhibitors were examined using bifunctional recombinant *Tb*DHFR-TS, all of which, apart from FdUMP, can be categorised as antifolates. Overall, *T*. *brucei* DHFR appears to be more exploitable in terms of selective inhibition over the human homologue than TS.

In the current study, the only molecules to possess picomolar *K*_*i*_ values against DHFR were trimetrexate and methotrexate, consistent with a previous report [51]. Maximal methotrexate potency *in vivo* was found to be dependent on the absence of thymidine from *T*. *brucei* growth media, thus confirming that thymineless death is part of its mode of action. However, the inability of thymidine to completely reverse methotrexate toxicity suggests that additional targets also exist beyond DHFR and TS. One likely candidate is pteridine reductase 1 (*K*_i_^app^ 11.1 nM) [51], another validated target in these parasites [52,53]. Compared to methotrexate, the pronounced potency of trimetrexate against DHFR is not reflected against whole cells likely due to the fact that trimetrexate is lipophilic and lacks a terminal glutamyl moiety for polyglutamylation and increased retention in the trypanosome (Fig 6).

Trimethoprim and pyrimethamine are both competitive inhibitors with intermediate potency against *T*. *brucei* DHFR. Our *K*_*i*_ values are 30- to 60-fold higher than first reported [13], but consistent with a subsequent report [48]. For these diaminopyrimidines, this translates to modest selectivity at best between parasite and host DHFR, compared to the > 100,000-fold selectivity of the antibacterial trimethoprim between human and *E*. *coli* DHFR [54]. A variety of novel diaminopyrimidines reported by Chowdhury *et al*. have been shown to be potent against *T*. *brucei* DHFR with nanomolar *K*_i_ values and selectivity up to 610-fold over the human enzyme [48]. However, toxicity and poor *in vivo* potency were limitations associated with these compounds. Both trimethoprim and pyrimethamine displayed a marked drop-off in potency from target to cell, when cultured in a medium deficient in thymidine and folate. Like trimetrexate these are lipophilic antifolates and do not contain a terminal glutamyl moiety. The addition of thymidine had little impact on cell potency of pyrimethamine or trimethoprim, implying that thymineless death is not their sole mode of action. This is consistent with a previous report which showed that the potency of pyrimethamine was not significantly affected by knocking out *dhfr-ts* [6].

With regards to antifolates possessing selectivity for TS over DHFR, compounds with a primary amine at position 4 correlated with stronger inhibition of DHFR, as was expected from previous reports, whereas a carbonyl substituent in this position is known to favour TS inhibition by means of additional hydrogen bonding provided by the oxygen atom [55]. TS-targeted antifolates also frequently include a terminal glutamyl moiety which is polyglutamylated *in vivo* by the enzyme folylpolyglutamyl synthetase (FPGS) [56]. This results in tighter binding to TS, with little effect on DHFR, and improved cellular retention. Although a candidate gene for FPGS is present in the *T*. *brucei* genome [57], it has not yet been studied in this species; however, in the related trypanosome *Leishmania*, intracellular folates possess on average 3–5 glutamates [58], thus this is likely also the case in *T*. *brucei*. Of the TS-targeted antifolates tested, the only compound which cannot exploit polyglutamylation was nolatrexed. Without the need for polyglutamylation, nolatrexed was found to be 10-fold TS selective; however, *in vivo* data showed moderate whole cell potencies. By comparison, the monoglutamate forms of raltitrexed and pemetrexed, generally thought of as TS-targeted antifolates in their polyglutamate forms, were found to be more potent against DHFR than TS, as has previously been observed with the monofunctional human enzymes [11,12]. Raltitrexed and pemetrexed are more potent against whole parasites than they are against DHFR or TS suggesting that polyglutamylation is likely to occur inside *T*. *brucei*. Off-target effects can be discounted since the trypanocidal action of these drugs is completely abrogated by thymidine and suggests that these inhibitors are likely to be TS-specific *in vivo*. Further work is required to substantiate this hypothesis.

Based on our observations regarding the inhibition of DHFR and TS by known antifolates, and their potency *in vivo* with regards to thymidine bypass, it is clear that additional targets must exist in *T*. *brucei*. Given their structural similarity to folate metabolites, possible alternative targets would be the FPGS, the glycine cleavage system, methionine synthase (Fig 1) or the bifunctional *N*^5^,*N*^10^-methylenetetrahydrofolate dehydrogenase-*N*^5^,*N*^10^-methenyltetrahydrofolate cyclohydrolase (DHCH). If the inhibition of one or more of these proposed targets can be identified then they could potentially be explored as alternative targets for the disruption of folate metabolism, which could be of interest for future drug discovery should *Tb*DHFR-TS prove difficult to exploit.

## Supporting Information

S1 Fig Confirmation of recombinant TS activity by complementation of TS-deficient (*thyA*^-^) *E*. *coli*. (PPTX)

S2 Fig Effect of pH and ionic strength on Tsf-*Tb*DHFR-TS activity. (PPTX)

S1 Table Cloning primers. (DOCX)

S2 Table Composition of HMI9T and trypanosome base medium (TBM). (DOCX)
