# Supplementary material for: Trypanosoma brucei DHFR-TS Revisited: Characterisation of a Bifunctional and Highly Unstable Recombinant Dihydrofolate Reductase-Thymidylate Synthase
Source: PLoS Negl Trop Dis. 2016 May 13;10(5):e0004714. doi: 10.1371/journal.pntd.0004714 (PMC4866688; doi:10.1371/journal.pntd.0004714)
Supplement: S1 Fig — (PPTX) [file pntd.0004714.s001.pptx]

## Slide 1
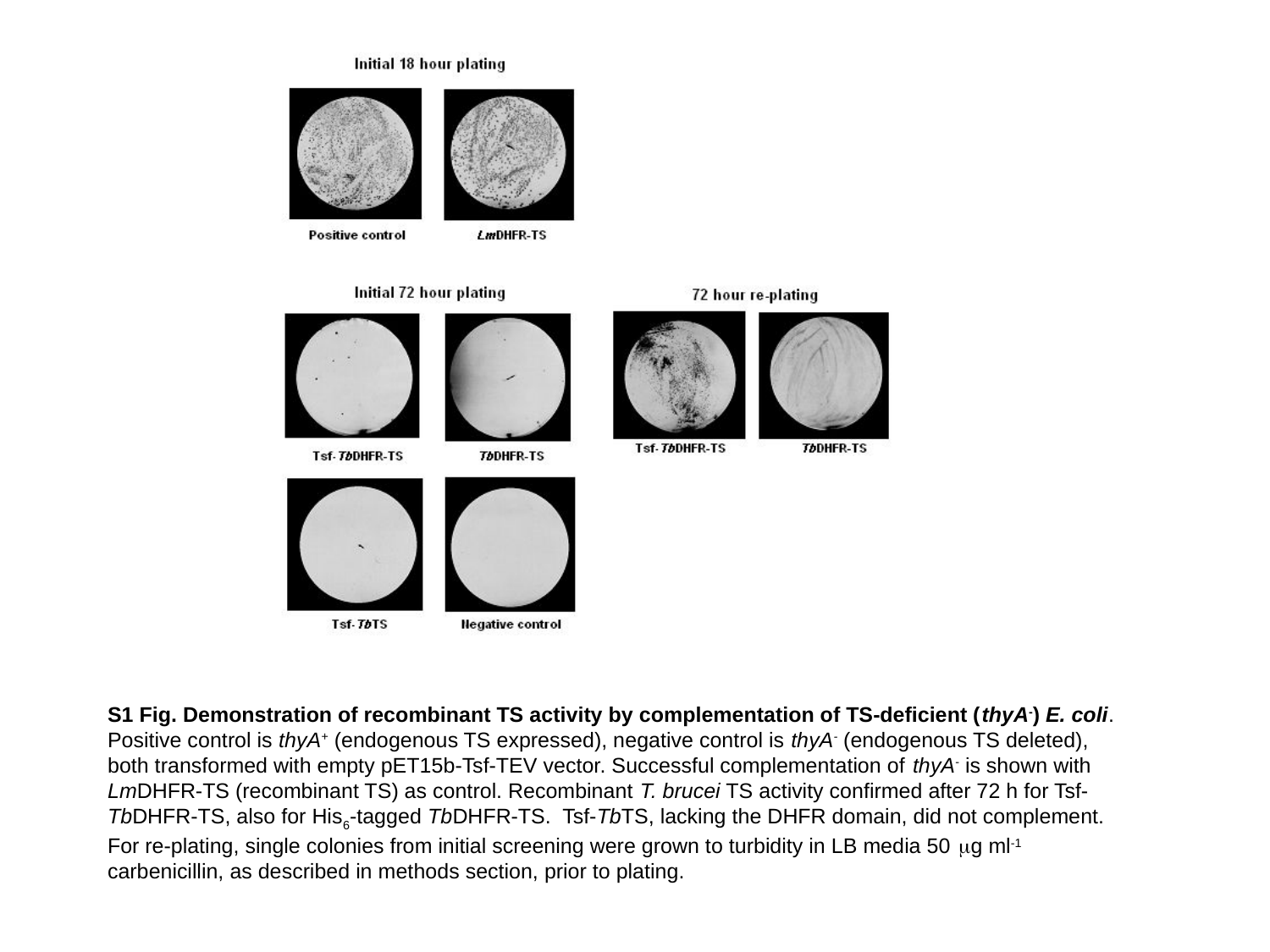

S1 Fig. Demonstration of recombinant TS activity by complementation of TS-deficient (thyA-) E. coli. Positive control is thyA+ (endogenous TS expressed), negative control is thyA- (endogenous TS deleted), both transformed with empty pET15b-Tsf-TEV vector. Successful complementation of thyA- is shown with LmDHFR-TS (recombinant TS) as control. Recombinant T. brucei TS activity confirmed after 72 h for Tsf-TbDHFR-TS, also for His6-tagged TbDHFR-TS. Tsf-TbTS, lacking the DHFR domain, did not complement. For re-plating, single colonies from initial screening were grown to turbidity in LB media 50 mg ml-1 carbenicillin, as described in methods section, prior to plating.
