# Supplementary material for: Trypanosoma brucei DHFR-TS Revisited: Characterisation of a Bifunctional and Highly Unstable Recombinant Dihydrofolate Reductase-Thymidylate Synthase
Source: PLoS Negl Trop Dis. 2016 May 13;10(5):e0004714. doi: 10.1371/journal.pntd.0004714 (PMC4866688; doi:10.1371/journal.pntd.0004714)
Supplement: S2 Fig — (PPTX) [file pntd.0004714.s002.pptx]

## Slide 1
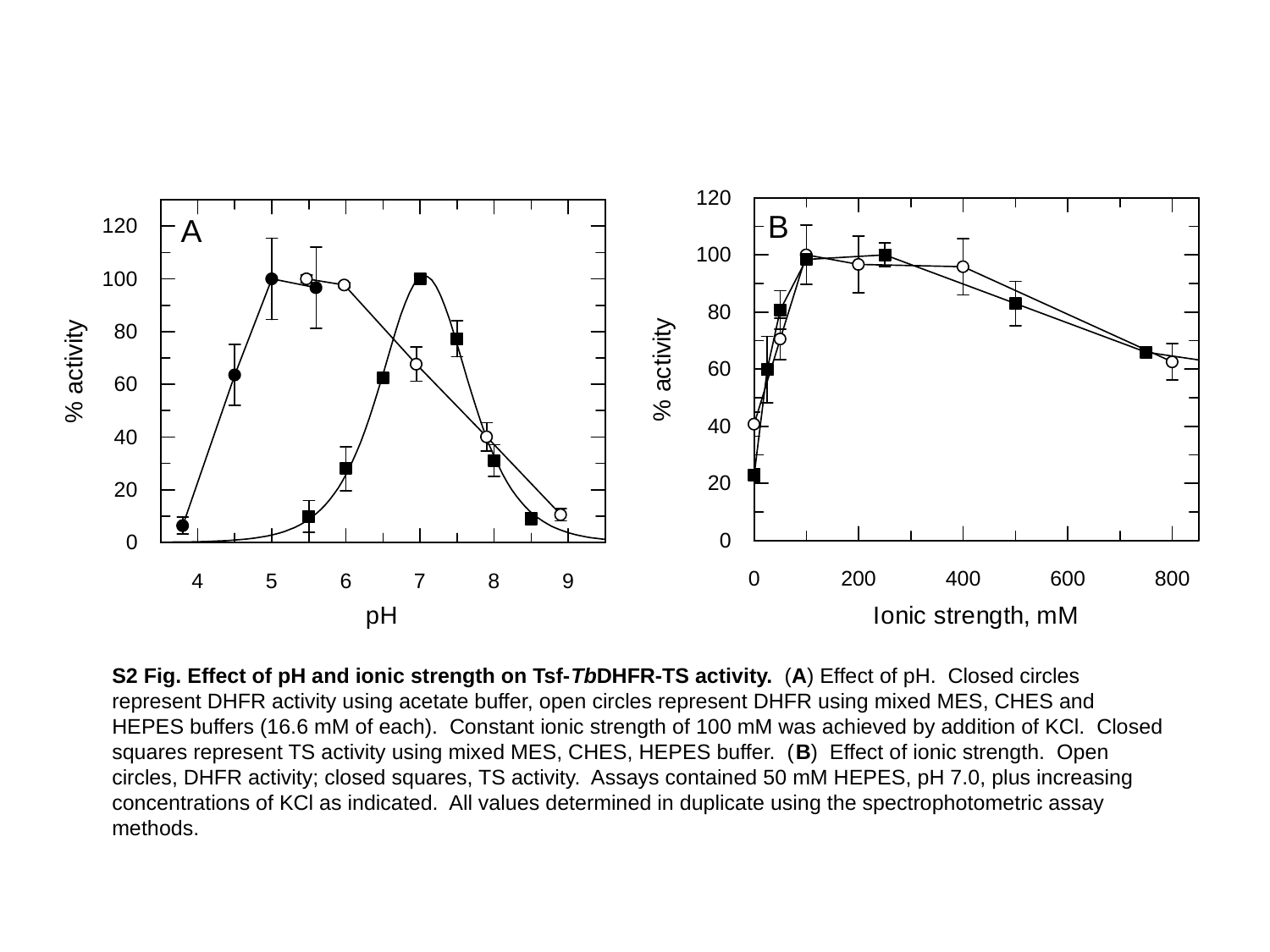

B
A
S2 Fig. Effect of pH and ionic strength on Tsf-TbDHFR-TS activity. (A) Effect of pH. Closed circles represent DHFR activity using acetate buffer, open circles represent DHFR using mixed MES, CHES and HEPES buffers (16.6 mM of each). Constant ionic strength of 100 mM was achieved by addition of KCl. Closed squares represent TS activity using mixed MES, CHES, HEPES buffer. (B) Effect of ionic strength. Open circles, DHFR activity; closed squares, TS activity. Assays contained 50 mM HEPES, pH 7.0, plus increasing concentrations of KCl as indicated. All values determined in duplicate using the spectrophotometric assay methods.
