# Supplementary material for: Trypanosoma brucei DHFR-TS Revisited: Characterisation of a Bifunctional and Highly Unstable Recombinant Dihydrofolate Reductase-Thymidylate Synthase
Source: PLoS Negl Trop Dis. 2016 May 13;10(5):e0004714. doi: 10.1371/journal.pntd.0004714 (PMC4866688; doi:10.1371/journal.pntd.0004714)
Supplement: S1 Table — (DOCX) [file pntd.0004714.s003.docx]

**S1 Table. Cloning primers**

Cloning primers used for generating constructs. Restriction endonuclease sites are underlined.

| **Primer name** | **Primer sequence** |
| --- | --- |
| *EcTsf*_s  *EcTsf* _as  *TbDHFR-TS*_s  *TbDHFR-TS*_as | 5´ GCG CCC ATG GCT GAA ATT ACC GCA 3´  5´ GC GCC CAT GGT AGA CTG CTT GGA CAT 3´  5´ GC GCG GAT CCG ATG CTC AGC TTT ACG 3´  5´ GCG CGG ATC CTA CAC CGC CAT CTC 3´ |
| *TbTS*_s | 5´ GC GCG GAT CCG AGC GAA GAG GAG CAG 3´ |
| *TbTS*_as | 5´ GCG CGG ATC CTA CAC CGC CAT CTC 3´ |
| *TbDHFR_*mut_s | 5´ G AAG CTT GTC CCC CGA TAA AGC GAA GAG GAG CAG 3´ |
| *TbDHFR_*mut_as | 5´ CTG CTC CTC TTC GCT TTA TCG GGG GAC AAG CTT C 3´ |
