# Supplementary material for: Trypanosoma brucei DHFR-TS Revisited: Characterisation of a Bifunctional and Highly Unstable Recombinant Dihydrofolate Reductase-Thymidylate Synthase
Source: PLoS Negl Trop Dis. 2016 May 13;10(5):e0004714. doi: 10.1371/journal.pntd.0004714 (PMC4866688; doi:10.1371/journal.pntd.0004714)
Supplement: S2 Table — (DOCX) [file pntd.0004714.s004.docx]

**S2 Table. Composition of HMI9T and trypanosome base medium (TBM).**  Key differences are highlighted in red.

| **COMPONENTS** | **HMI9T (mM)** | **TBM (mM)** |
| --- | --- | --- |
| **Amino acids** |  | |
| Glycine | 0.400 | 0.400 |
| L-Alanine | 0.281 | 0.281 |
| L-Arginine | 0.398 | 0.398 |
| L-Asparagine | 0.167 | 0.167 |
| L-Aspartic acid | 0.226 | 0.226 |
| L-Cystine 2HCl | 0.381 | 0.381 |
| L-Glutamic Acid | 0.510 | 0.510 |
| L-Glutamine | 4.00 | 2.00 ^a^ |
| L-Histidine | 0.200 | 0.200 |
| L-Isoleucine | 0.802 | 0.802 |
| L-Leucine | 0.802 | 0.802 |
| L-Lysine hydrochloride | 0.798 | 0.798 |
| L-Methionine | 0.201 | 0.201 |
| L-Phenylalanine | 0.400 | 0.400 |
| L-Proline | 0.348 | 0.348 |
| L-Serine | 0.400 | 0.400 |
| L-Threonine | 0.798 | 0.798 |
| L-Tryptophan | 0.0784 | 0.0784 |
| L-Tyrosine disodium salt | 0.462 | 0.462 |
| Valine | 0.803 | 0.803 |
| **Vitamins** |  | |
| Biotin | 0.0000533 | 0.0000533 |
| Choline chloride | 0.0286 | 0.0286 |
| D-Calcium pantothenate | 0.00839 | 0.00839 |
| Folic Acid | 0.00807^b^ | 0^c^ |
| i-Inositol | 0.0400 | 0.0400 |
| Niacinamide | 0.0328 | 0.0328 |
| Pyridoxal hydrochloride | 0.0196 | 0.0196 |
| Riboflavin | 0.00106 | 0.00106 |
| Thiamine hydrochloride | 0.0119 | 0.0119 |
| Vitamin B12 | 0.0000096 | 0.0000096 |
| **Salts** |  |  |
| Calcium Chloride (CaCl_2_) | 1.49 | 1.49 |
| Potassium Nitrate (KNO_3)_ | 0.000752 | 0.000752 |
| Magnesium Sulphate (MgSO_4_) | 0.814 | 0.814 |
| Potassium Chloride (KCl) | 4.40 | 4.40 |
| Sodium Bicarbonate (NaHCO_3_) | 36.00 | 36.00 |
| Sodium Chloride (NaCl) | 77.59 | 77.59 |
| Sodium Phosphate | 0.906 | 0.906 |
| Sodium Selenite (Na_2_SeO_3_.5H_2_O) | 0.0000658 | 0.0000658 |
| **Other components** |  |  |
| D-Glucose (Dextrose) | 25.00 | 25.00 |
| HEPES | 25.03 | 25.03 |
| Phenol Red | 0.0399 | 0.0399 |
| Sodium Pyruvate (total) | 1.0 (2.0)^d^ | 1.0 (2.0)^d^ |
| **Additional supplements** |  |  |
| Bathocuproine sulphonate | 0.05 | 0.05 |
| Cysteine | 1.5 | 1.5 |
| Hypoxanthine | 1.0 | 1.0 |
| Thioglycerol | 0.2 | 0 |
| 2-Mercaptoethanol | 0 | 0.2 |
| Pyruvate | 1.0 (see above) | 1.0 (see above) |
| Thymidine | 0.16 | 0 ^c^ |
| Foetal bovine serum (%) | 10 | 10 |
| Serum Plus (%) | 10 | 0 |

1. As GIBCO ® GlutaMAX™, L-alanyl-L- glutamine, a dipeptide substitute for L-glutamine
2. From Iscove’s Modified Dulbecco’s Media and 10% Serum Plus, excluding any contribution from FBS
3. Excluding any contribution from FBS
4. Values in parentheses include additional supplements
